# Exploration of barriers to treatment for patients with eating disorders in Chile

**DOI:** 10.1186/s40337-024-01104-x

**Published:** 2024-10-12

**Authors:** Felipe Castañeda, Jaime Cerda, Raúl Jara, Francisca Riestra, Pascuala Urrejola, Melina Vogel, María Elena Gumucio, Verónica Irribarra, Jorge Álvarez, María Alejandra Díaz, Paula Kompatzki, Daniela Costa

**Affiliations:** 1 School of Medicine, Faculty of Medicine, Pontificia Universidad Católica de Chile, Santiago, Chile; 2 Department of Public Health, Faculty of Medicine, Pontificia Universidad Católica de Chile, Santiago, Chile; 3 Department of Psychiatry, Faculty of Medicine, Pontificia Universidad Católica de Chile, Santiago, Chile; 4 Department of Psychiatry, Complejo Asistencial Doctor Sótero del Río, Puente Alto, Santiago Chile; 5 Eating Disorders Unit, UC Christus Health Network, Santiago, Chile; 6 Department of Pediatrics, Faculty of Medicine, Pontificia Universidad Católica de Chile, Santiago, Chile; 7 Department of Nutrition, Diabetes and Metabolism, Faculty of Medicine, Pontificia Universidad Católica de Chile, Santiago, Chile

**Keywords:** Eating disorders, Access to treatment, Early intervention, Public health, Chile

## Abstract

**Background:**

Eating disorders (EDs) are associated with high morbidity and mortality, affecting predominantly young people and women. A delay in starting treatment is associated with chronic and more severe clinical courses; however, evidence on barriers and facilitators of access to care in Latin America is scarce. We aimed to identify barriers and facilitators of ED treatment in Chile from the perspective of patients, relatives, and health professionals.

**Methods:**

Qualitative approach through semi-structured interviews with patients, their relatives, and health professionals. Participants were recruited from two ED centers in Santiago, Chile (one public and one private). Analysis was mainly based on Grounded Theory, using MAXQDA software.

**Results:**

40 interviews were conducted (n = 22 patients, 10 relatives, and 8 health professionals). The mean age of patients was 21.8 years, while the mean duration of untreated ED was 91.4 months (median 70 months). Five categories emerged with intersections between them: patient (P), family and social environment (FSE), health professionals (HP), healthcare system (HCS), and social and cultural context (SCC). Relevant barriers appeared within these categories and their intersections, highlighting a lack of professional knowledge or expertise, cultural ignorance or misinformation regarding EDs, and patient’s ego-syntonic behaviors. The main facilitators were patients’ and relatives’ psychoeducation, recognition of symptoms by family members, and parents taking the initiative to seek treatment.

**Conclusions:**

This study provides information regarding access to treatment for patients living with EDs in Chile. A practical public health approach should consider the multi-causality of delay in treatment and promoting early interventions.

**Plain English Summary:**

Eating disorders (EDs) may severely affect the daily functioning of people enduring them. A delay in starting treatment is associated with a disease that is more difficult to treat. To our knowledge, there are no published studies carried out in Latin America exploring factors influencing treatment initiation in EDs patients. This study aimed to identify facilitators of and barriers to treating patients with EDs in Chile. We interviewed patients (n = 22), their relatives (n = 10), and health professionals (n = 8) from a private and a public center in Santiago, Chile. Our analysis showed that the main barriers to starting treatment were the lack of professional knowledge in ED, the monetary cost of illness, and cultural misinformation. Facilitators were related to the role of the family in recognizing and addressing the disease while being educated in EDs by professionals. This study helps to provide data about treatment access in developing countries. While facilitators and barriers were similar to others reported in the literature, the untreated ED’s duration was longer. It is essential to address these barriers to provide access to treatment more efficiently and prevent severe and enduring forms of disease.

**Supplementary Information:**

The online version contains supplementary material available at 10.1186/s40337-024-01104-x.

## Background

Eating disorders (EDs) have the highest mortality among mental illnesses [1], due to impairment of physical health or psychological complications [2]. Paradoxically, most individuals with EDs do not seek treatment [3, 4]. A delay in starting treatment for an ED is a marker for a worse prognosis [5] and can lead to an ‘entrenched’ form of the disease, which is often more challenging to treat [3]. Evidence shows that early intervention leads to better outcomes in patients with EDs [6, 7]. Moreover, it reduces the duration of the untreated eating disorder (DUED), which refers to the time elapsed between the onset of symptoms and the initiation of appropriate treatment. Understanding the barriers and facilitators to access ED treatment is essential for identifying factors that contribute to prolonged DUED and may provide insights into the delays patients face before receiving appropriate care.

Current research suggests that stigma, lack of knowledge about mental disorders, negative past experiences with professionals, and lack of support from the social environment, among other barriers, may hinder access to treatment in EDs [8]. However, the literature remains scant and lacks data on factors that influence treatment initiation in developing countries.

According to the Global Burden of Disease Study 2019 [9], Chile has the highest prevalence of both anorexia nervosa (AN) and bulimia nervosa (BN) in Latin America (0.09% and 0.27%, respectively) [10]. Treating patients with EDs involves a high economic cost for families and the system as a whole [2, 11]. Moreover, patients with EDs may present a broad spectrum of complications, which tend to be particularly serious in patients with AN [12]. Existing literature shows that a more severe clinical course may be associated with a chronic ED [13]. A study of 41 patients treated in a specialized ED unit in Chile found that the clinical severity of the disorder was associated with a delay in finding treatment [14]. Thus, the need to study the factors that affect access to treatment is important, particularly in countries where access to mental health care is not widespread [14].

There is a need to address EDs in Latin America, as there are few published studies regarding epidemiology, cultural factors [15], role of ethnicity or culture-related differences as risk factors for EDs [10]. This knowledge gap is even greater when referring to barriers to access to treatment. To our knowledge, no studies have yet been published about factors that influence treatment initiation in ED patients from Latin American countries [16].

Considering that EDs are identified in primary care or pediatric consultations [17], it is necessary to know the availability of referral services. Also, several studies have evaluated the knowledge and skills of health professionals in diagnosing and treating patients with EDs [18–22].

The Chilean healthcare system has a mixed financing scheme, with both public and private sources. The public system is financed through state funds and mandatory individual contributions, while the private system is financed by individual insurance based on the risk of the insured [23]. High-risk and poorer individuals are more prevalent in the public system, increasing inequalities by placing increased demand on limited resources [24]. On the other hand, the private system is highly susceptible to financial catastrophe due to the high out-of-pocket expenditures for families [24]. Moreover, the Chilean healthcare system risks collapsing entirely following increased clashes between the national judiciary and private insurers [25]. We included the experiences of patients and families from private and public healthcare to address system-specific facilitators and barriers.

This study aimed to identify barriers and facilitators of access to treatment for patients with EDs in Chile from the perspective of patients, family members, and health professionals. The theoretical framework of this study used evidence from other countries because current data from Latin America remains poor on treatment initiation and first contact in EDs.

Additionally, replicating a recent German study [26], this investigation quantitatively estimated the (DUED).

## Methods

### Design and setting

The study has a qualitative, multi-informative design. Data was collected from semi-structured interviews (n = 40) with participants from both a private Chilean healthcare network (n = 30) and a public hospital (n = 10). A specific interview script was designed for the three study groups: patients, family members, and health professionals. We worked primarily under a Grounded Theory approach [27, 28], reaching theoretical saturation after analyzing sufficiently heterogeneous collected data. Theoretical saturation is defined as the point at which no new themes or insights are being observed in the data [29].

Data collection and analysis were both conducted following the Consolidated Criteria for Reporting Research (COREQ) recommendations [30]. We also followed the Standards for Reporting Qualitative Research [31]. These checklists are included in a supplementary file.

### Participants

Heterogeneous patients regarding gender, age, socioeconomic status, city of residence, migrant status, and specific ED were included until a saturation point was reached. Participants were initially chosen by purpose, which meant that they were purposively chosen to ensure a diverse demographic representation, including various age groups, genders, socioeconomic and geographical backgrounds. As interviews were conducted and transcribed, a data matrix was created in Excel; this helped to systematize information and generate selection criteria for the following participants. With the help of specialized ED teams, it was possible to facilitate the targeted search for patients whose “a priori” information could help fill content gaps. Thus, theoretical saturation was reached with 40 interviews, which were distributed among 22 patients, 10 family members, and 8 health professionals. The sample sizes for each group were not determined in advance, but based on the need to capture a comprehensive range of perspectives until no new information was being discovered. Nevertheless, we wanted to focus primarily on the patient experience, so we assured to keep a majority of interviews with patients rather than with family members and health professionals.

The patients’ inclusion criteria were being at least 14 years old, diagnosed with an eating disorder according to DSM-5, having started treatment for the first time within the last two years, and with at least five or more outpatient treatment sessions, or seven days of inpatient treatment. Exclusion criteria were severe psychiatric or somatic symptoms that may affect participation, having a previous treatment for the ED, and active drug use. The two-year limitation for starting treatment was to reduce the possible effect of recall bias [32]. To exclude early treatment dropouts, five outpatient sessions or seven days of inpatient treatment were required [33]. During the interviews, patients were asked about a family member and a healthcare provider who were significant in their treatment, and subsequently, they were contacted. No exclusion criteria were previously defined for family members and health professionals, so, for instance, adolescent siblings were allowed to participate.

This study was a thesis for a master’s degree in public health and received approval from the ethics committee of the Pontificia Universidad Católica de Chile and the Southeast Metropolitan Health Service in Santiago. Additionally, before starting recruitment, we obtained authorization from San Carlos de Apoquindo Clinic and Dr. Sótero del Río Hospital medical management. Informed consent was obtained from all participants. For those under 18 years of age, parental consent and assent from the minor was obtained.

### Data collection

Patients were recruited from each medical center by their health professionals. If they accepted participation, they were contacted by the lead researcher and sent the informed consent form, after which an interview was scheduled. Compliance with inclusion criteria and exclusion criteria was verified. Relatives and health professionals were contacted and interviewed after patients named them as significant for their treatment and had given their consent.

Interviews were conducted privately via Zoom or in-person by the leading researcher, lasting between 30 and 90 min. Once the interviews and their corresponding audio files were completed, they were stored on a password-protected electronic platform. The verbatim transcription of the recordings was made using Microsoft Word by the leading researcher and two research assistants. Transcripts were then uploaded to MAXQDA.

Specific interview scripts were designed for patients, relatives, and health professionals. The scripts were designed to obtain socio-demographic information from participants and to identify facilitators and barriers.

An attempt to quantitatively estimate the DUED was made for patients. It was calculated by subtracting the treatment start date from the onset of symptoms date (defined as the self-reported first occurrence of significant body image dissatisfaction or dieting behavior). If the patient remembered the specific month and year of the beginning of symptoms, that date was recorded. However, if they did not recall the exact date, an average of two approximate dates was calculated (for example, “between January and July 2011” was recorded as April 2011). The treatment starting date was confirmed by the healthcare provider.

The script was based on a German study [26, 33]. The leading researcher contacted Dr. Antje Gumz before starting the study and after its completion. Methodological aspects were adapted to the local context.

### Data analysis

Analysis was carried out in Microsoft Excel and MAXQDA, by FC and FR, co-occurring with data collection.

There were three modes of coding the interviews: open, axial, and selective coding. As the interviews were conducted, the categories of analysis emerged. An attempt was made to generate a tree of codes that adequately represented the content of the interviews, which was completed in the analysis’s final stages. The definitive model included five main categories with intersections between them.

The specific value of each intersection between categories was emphasized. Thus, for example, the codes that pointed to the interaction between the patient (P) and family environment (FSE) were grouped at the intersection “FSE + P”.

The positive or negative value that participants gave to each factor mentioned during the interview was carefully evaluated to identify facilitators and barriers. Therefore, depending on the context, some aspects could function both as facilitators and barriers (for example, a positive experience with a health professional versus a negative one). An F (facilitator) or B (barrier) was added to each code for being either a facilitator or barrier and was grouped into a model category.

The description of sociodemographic variables and the estimation of DUED were conducted using descriptive analysis with measures of central tendency, dispersion, and frequencies through Excel.

During the analysis process, an audit of five interview recordings was performed by an external reviewer as part of a reliability check.

## Results

### Sociodemographic characteristics

Overall, 40 interviews were conducted: 22 patients, 10 relatives, and 8 health professionals. 35 were conducted using Zoom, while 5 were in-person. 90% of the interviewees were women (n = 36), while 10% were male (n = 4). No participant required psychological support during the interview process. No invited people refused to participate or dropped out.

Table 1 shows the demographic and clinical characteristics of patients, relatives, and health professionals. Diagnostic criteria and BMI upon entering treatment were reported by the professional.

**Table 1:** Sample characteristics

Demographic or clinical characteristic	Sample characteristics (n = 40)
*Patients (n = 22)*	
Gender	Female: n = 21
	Male: n = 1
Geographical origin	Northern region of Chile: n = 1
	Central region of Chile: n = 18
	Southern region of Chile: n = 3
Initial treatment	Outpatient: n = 17
	Inpatient: n = 5
Living with parents	With both parents: n = 11
	Only with mother: n = 6
	With neither parent: n = 5
Highest education level completed (personal)	Primary education: n = 8
	Secondary education: n = 10
	Short-cycle tertiary education: n = 2
	Bachelor’s degree: n = 2
Highest education level completed (parents)	Secondary education: n = 9
	Bachelor’s degree: n = 13
Health insurance	Private = n = 15
	Public: n = 7
Migrant status	Yes: n = 1
Identification with indigenous group	Yes: n = 1
Eating disorder diagnosis	Anorexia nervosa: n = 10
	Restrictive subtype: n = 7
	Purgative subtype: n = 3
	Bulimia nervosa: n = 4
	OSFED: n = 3
	Atypical anorexia: n = 3
	Binge-eating disorder: n = 2
	UFED: n = 2
	ARFID: n = 1
Age (at the time of interview)	M = 21.8 years (SD = 7.46)
BMI at the start of treatment	M = 19.67 kg/m^2^ (SD = 5.05)
DUED	M = 91.41 months (SD = 82.28)
	Median = 70 months (Range: 8 – 298)
*Relatives (n = 10)*	
Relationship with patient	Relative 1: Mother of a 16-year-old adolescent
	Relative 2: Mother of a 19-year-old adolescent
	Relative 3: Mother of a 19-year-old adolescent
	Relative 4: Mother of a 16-year-old adolescent
	Relative 5: Mother of a 16-year-old adolescent
	Relative 6: Mother of a 16-year-old adolescent
	Relative 7: Mother of a 17-year-old adolescent
	Relative 8: Mother of a 33-year-old adult
	Relative 9: Sister of a 22-year-old adult
	Relative 10: Husband of a 44-year-old adult
Geographical origin	Central region of Chile: n = 7
	Southern region of Chile: n = 3
Age (at the interview)	M = 51.7 years (SD = 5.19)
*Health professionals (n = 8)*	
Gender	Female: n = 6
	Male: n = 2
Profession	Physician: n = 4
	Psychiatrist: n = 3
	Pediatrician: n = 1
	Nutritionist: n = 2
	Psychologist: n = 2
Specialization in ED	Yes: n = 6
	No: n = 2
Working in healthcare subsystems	Only in private system: n = 6
	Both in public and private: n = 2
Age (at the time of interview)	M = 39.9 years (SD = 10.33)

**Fig. 1:**
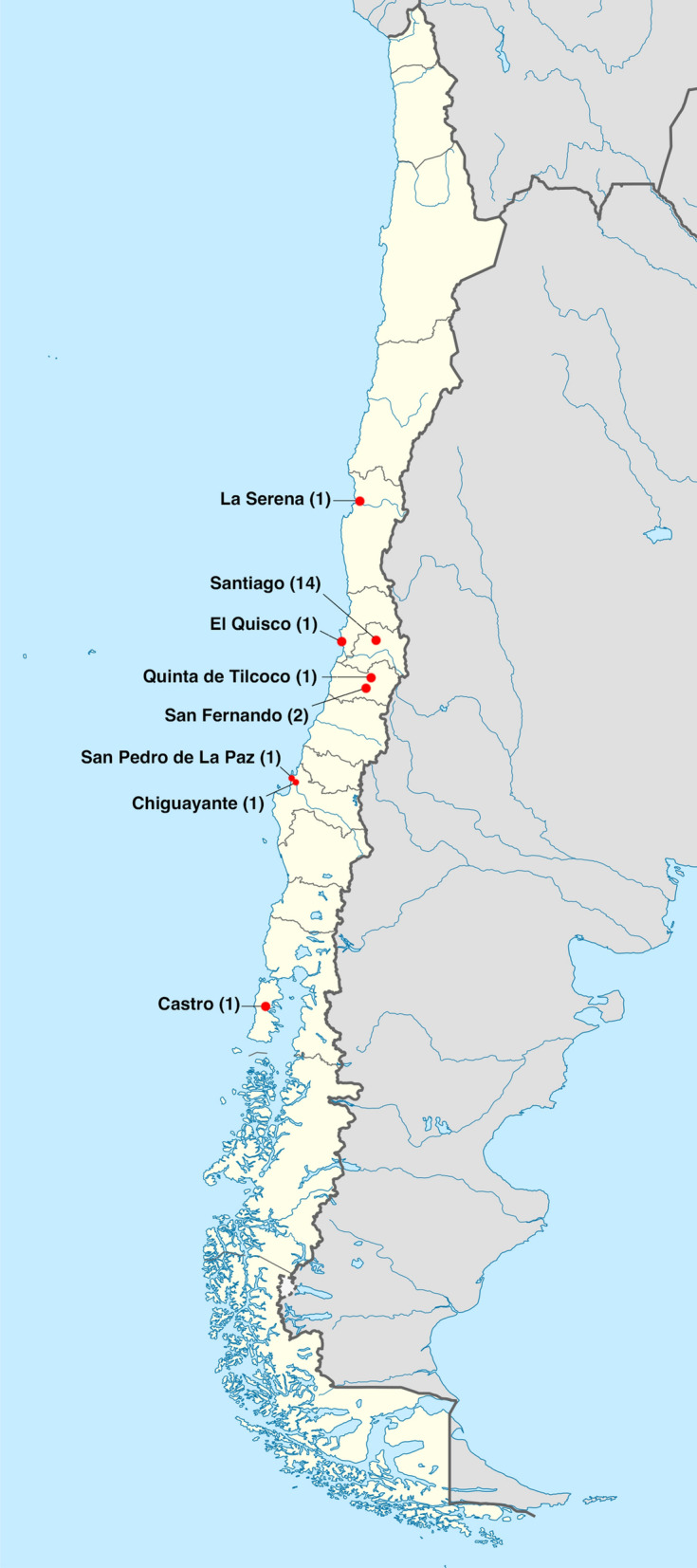
Geographical origin of the patient sample within Chile

The geographic location of patients may serve to illustrate the physical inaccessibility of specialized ED services in Chile (Fig. 1). While 14 of 22 patients lived in Santiago at the time of the interview, all healthcare services included in the study were in Santiago. That means, 8 of 22 patients received a long-distance treatment.

### Emerging categories, facilitators, and barriers

**Fig. 2:**
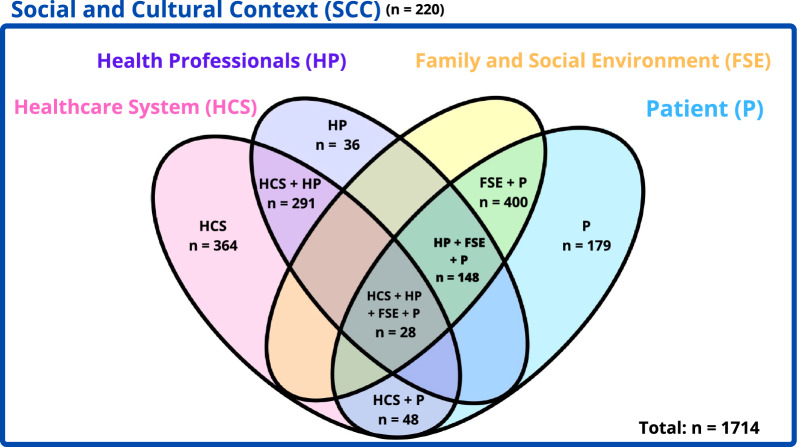
Emerging categories model and number of codes

The final model included 1714 codes, which were divided into 5 main categories (Fig. 2): patient (P), family and social environment (FSE), health professionals (HP), healthcare system (HCS), and social and cultural context (SCC).

Tables 2 and 3 show 15 of the most important barriers and facilitators respectively. Also, to favor the replicability of this type of research, the results are presented similarly to Kästner et al. [26].

**Table 2:** Top 15 barriers to treatment initiation

Barriers	Category^a^	Number of codes	Number of interviews (%)^b^
Lack of professional knowledge or expertise	HP–HCS	140	34 (85)
Monetary cost of treatment	HCS	104	31 (77.5)
Cultural ignorance or misinformation	SCC	80	32 (80)
Patient’s ego-syntonic behaviors	P	101	31 (77.5)
Family does not recognize signs or symptoms	FSE–P	79	29 (72.5)
Lack of specialists or teams for ED treatment	HCS	66	28 (70)
Stigma or stereotypes about ED	SCC	41	22 (55)
Geographic location of treatment services	SCC	63	19 (47.5)
Normalization of maladaptive eating patterns	SCC	34	21 (52.5)
Family does not understand or support	FSE–P	36	18 (45)
Not being able to talk about (my) eating problems	P–FSE–HP	30	21 (52.5)
Long-standing ED	P	31	18 (45)
Lack of multidisciplinary team in (my) treatment	HP–HCS	29	17 (42.5)
Long and/or difficult therapeutic process	P–FSE	35	13 (32.5)
Not knowing where or who to turn to	P–FSE–HP–HCS	26	17 (42.5)
Positive value of society on female thinness	SCC	29	16 (40)

^a^Abbreviations: *HP* health professionals; *HCS* healthcare system; *SCC* social and cultutral context; *FSE* family and social environment; *P* patient. ^b^Percentage of all interviews (n = 40)

**Table 3:** Top 15 facilitators of treatment initiation

Facilitators	Category^a^	Number of codes	Number of interviews (%)^b^
Psychoeducation for patients and their families	HP–P–FSE	87	27 (67.5)
Parents taking the initiative to find treatment	FSE–P	51	27 (67.5)
Recognition of symptoms by the family	FSE–P	48	27 (67.5)
Understanding or support of the family group	FSE–P	66	25 (62.5)
Referral or recommendation to treatment	HCS–HP	44	25 (62.5)
Presence of multidisciplinary team in (my) treatment	HP–HCS	41	25 (62.5)
Clinical worsening	P–HCS	48	23 (57.5)
Insight or patient’s ED awareness	P	42	24 (60)
Professional knowledge or expertise	HP–HCS	31	19 (47.5)
Having financial resources for access	FSE–P	25	19 (47.5)
Being able to talk about (my) eating problems	P–FSE–HP	25	18 (45)
There’s a greater awareness or visibility of ED	SCC	27	15 (37.5)
Quick access from the referral	HCS	19	15 (37.5)
Inpatient treatment to access specialized management	HCS	23	14 (35)
Health professional empathy	HP	16	12 (30)

^a^Abbreviations: *HP* health professionals; *P* patient; *FSE* family and social environment; *HCS* healthcare system; *SCC* social and cultural context. ^b^Percentage of all interviews (n = 40)

### Main findings

1080 barriers and 634 facilitators (63% and 37%, respectively) were coded. This section addresses, using direct quotes, the practical impact or value of the main facilitators and barriers in the model.

### Patient

The main barrier within the patient category was ego-syntonic behaviors (101 codes; 77.5% of interviews). Ego-syntonic features of EDs entail symptoms of concealment, “not wanting to receive help” or “not wanting to get better.” One patient describes it as follows:

Patient 19*: “You don’t want to leave the disease. It’s your comfort zone and a life you’re used to. So, getting out of it is difficult, and even more so when you’ve been involved in this for so long.”*

This quote is also linked to one of the main barriers in the patient category, that is, the presence of a longstanding ED (31 codes; 45% of interviews). The most mentioned facilitator in this category was insight or awareness of the disease (42 codes; 60% of interviews). This facilitator could be seen as a logical counterpart to ego-syntonic behaviors, values, and feelings, but requires a complex process of personal understanding.

Patient 3*: “I wanted to get better. I truly wanted to get out of it. So, my perseverance made my parents look for places (for treatment).”*

### Intersection: patient and healthcare system

At this level, worsening symptoms are the main facilitator for starting treatment (48 codes; 57.5% of interviews). There was a heterogeneity of “worsening” symptoms within the interviews (for example, from a change in self-perceived mental health status to cardiorespiratory arrest). However, in all cases, it was a factor that favorably affected access to treatment.

Patient 17*: “If I hadn’t come to the emergency room due to malnutrition, we would never have started (treatment).”*

### Intersection: patient and family and social environment

Families were most frequently mentioned in the interactions between the patient and the environment, both as barriers and facilitators (see barriers 5 and 10 of Table 2 and facilitators 2–4 of Table 3). Family members are part of the patient’s care system, but at the same time, they may unintentionally contribute to the maintenance of the disease due to normalized attitudes.

*Patient 19: “They (my parents) realized that I wasn’t eating, and at one point, they even congratulated me for not eating so much because I was losing weight, and ‘that way I looked prettier.’ That’s why it was hard for me to tell them that ‘I feel bad eating’, and to find a specialist.”*

Therefore, it’s essential for health professionals to seek a therapeutic alliance with parents, empowering them to recognize the disease and to cope with it. The quote above addresses the family as a barrier, while the following two places it as a facilitating factor.

Patient 9*: “My mother is the one who always supports me and tells me that I must keep going, that together we can make it. That helped me the most to start treatment.”*

Patient 5*: “My friends didn’t notice, but my family did. They noticed my weight loss. My face looked tired, with dark circles. So, they worried more and more. I saw that my mom had a hard time when she saw me doing poorly. I think that influenced me to seek treatment.”*

### Intersection: patient, family and social environment, and health professionals

Clinicians, patients, and their families had different levels of understanding of EDs. Psychoeducation is a crucial facilitator for starting treatment and adherence (87 codes, 67.5% of interviews). It emphasizes, among other aspects, the need for prompt care due to the high risk of chronicity. The mother of a patient with AN relates the effect of psychoeducation as follows:

*Relative 3: “First, we didn’t do it well; We did not follow the meal plan as told, because I did not understand its importance. Until one day, the doctor told me, ‘You have to fight the disease.’ ‘You have to be very strict.’ And then it made sense. So, I told my husband, ‘The doctor told me that we have to fight, and the meal plan has to be followed.’ In other words, do not give way to this disease so that it cannot beat us. We beat it.”*

### Intersection: health professionals and healthcare system

The most mentioned barrier in the whole analysis was the professionals’ lack of knowledge or expertise (140 codes; 85% of interviews). Throughout the interviews, this lack of knowledge was brought up regarding visiting professionals who failed to identify the disease or who identified it but did not know how to manage it.

Patient 8:* “Ignorance about the disease. Not only from my parents but from all doctors in general. […] We visited many doctors, and no one knew anything.”*

*Patient 22: “I had a psychiatrist, but he misunderstood what was happening and didn’t know how to help me either. […] In fact, he told me that everything was fine if I had a BMI of 16 (kg/m*^*2*^*). If I reached a BMI of 14 (kg/m*^*2*^*), then I had to worry.”*

In some cases, patients and their families received treatment from more than one non-specialist treating professional who handled the ED inadequately. Also, this may be related to the apparent lack of medical education on EDs in undergraduate studies, which was mentioned by some professionals.

Health professional 5*: “There is also the phenomenon that non-specialists take these cases, and the literature says that not only is this not recommended, but it is iatrogenic. It can negatively affect that patient, even if the professional’s intention is good.”*

Health professional 3: *“I think that (medical) undergraduate formation is scarce in EDs. What I have seen in undergraduate programs is that, usually, there is a class in the fourth or fifth year on EDs, and that is it.”*

The most relevant facilitators at this intersection were treatment by a multidisciplinary team (41 codes; 62.5% of interviews) and a referral to a specialized ED unit (44 codes; 62.5% of interviews). Moreover, participants highlighted the role of specialists and primary care physicians in the early detection of EDs and timely referral to a specialized multidisciplinary team.

Relative 2*: “Thank God she had the chance to receive a multidisciplinary treatment. Not just the dietitian, the psychologist, or the physicians. But all of them working together.”*

*Patient 18: “I was referred from primary care. I told the doctor that I ate and vomited very often, almost daily. She got worried and referred me here with a psychiatrist.”*

### Healthcare system

In this sample, the Chilean healthcare system was perceived as offering more barriers than facilitators for patients with EDs. The high financial cost of treatment in the private system, which houses most ED specialists in the country, was consistently mentioned as a barrier (104 codes; 77.5% of interviews). This may be explained due to the need for a multidisciplinary team and short intervals between appointments, the lack of reimbursement by insurers, and the high cost of inpatient treatment.

Relative 1: “We are talking about four expensive specialists. Some people simply cannot access (treatment). I am spending around 600,000 pesos* a month.”

*Approximately 650 USD as of January 2024.

Patient 22*: “The cost of this is enormous. I feel hyper-privileged to do it because I couldn’t afford it, and my family is paying. My mom is trying to sell the house to help my sister with the cost of this because I’ve been hospitalized for two months. It is unthinkable. I don’t want to calculate what’s coming.”*

The geographic location of specialized health services is another barrier in the healthcare system (63 codes; 47.5% of interviews). Although this barrier was not mentioned by participants from Santiago, it was extensively brought up by patients from other regions.

Patient 9*: “We didn’t find many things in the Valparaíso Region. The only hope we had was Santiago. [...] Where I am (receiving treatment), many people must travel to Santiago. There are no other places to receive all this help.”*

Furthermore, this barrier is linked to another negative aspect of the healthcare system: the lack of specialists (66 codes; 70% of interviews).

Patient 6:* “Not many professionals specialize in these kinds of disorders. It was hard for me to find (a specialist). In fact, I could say that I never found one until I was hospitalized.”*

Health professional 4:* “Most of the specialists work in Santiago, which makes (the situation) more dramatic. These disorders require a degree of specialization, so, primary care teams or even mental health teams in other regions do not have the expertise to handle EDs correctly.”*

### Social and cultural context

Chilean society has yet to shift toward a culture that adequately recognizes and addresses EDs. Although in recent years there has been greater visibility towards EDs, and this was recognized as a facilitator in several interviews (27 codes; 37.5% of interviews), cultural ignorance and misinformation still exist (80 codes; 80% of interviews).

Relative 3*: “They treated us badly as parents because I felt they blamed us. The disease was very unknown. They couldn’t understand how we couldn’t feed our daughter.”*

Health professional 4*: “The nonrecognition shows that there is a lot of ignorance in our society regarding eating disorders.”*

Stigma (41 codes; 55% of interviews) and society’s positive value of female thinness (29 codes; 40% of interviews) are linked to misinformation. Those factors may perpetuate harmful behaviors and affect access.

Patient 2:* “The social stigma is that you must be thin. In the end, you may know you have an eating disorder, but you don’t want to get better because you feel more accepted in society. Nobody is going to judge you if you are skinny or if you fit into social standards.”*

## Discussion

This qualitative research explored facilitators and barriers to access to treatment in patients with EDs in Chile. Interviewing three different groups allowed us to identify factors that affect access to care. Furthermore, it was possible to establish that each group tended to mention more barriers and facilitators specific to their own perspective or field, which was also reported in the study by Kästner et al. [26]. For example, patients mostly described ego-syntonic behaviors as barriers, while health professionals emphasized the lack of knowledge or expertise. Meanwhile, the most important facilitators for families were the recognition of EDs and their own involvement in the treatment of their relatives.

Interestingly, each of the five main barriers found in this study represents a different category or intersection of the model proposed in the analysis, reinforcing the idea that the problem must be addressed from different angles. With regards to the five main facilitators, four emphasize the role of families. In EDs, family members play an essential role in the recovery of patients, an aspect that has been emphasized in other qualitative studies [34–36].

The barriers and facilitators found in the analysis are relatively similar to those found in other studies, such as the systematic review by Ali et al. [37] and the Scoping review by Nicula et al. [8]. The geographic barrier, which did not appear in these but was found in our study, was addressed in three studies from Australia and New Zealand [38–40]. Another barrier found in this study was the existence of a longstanding ED (45% of the interviews). Some data suggest that a prolonged clinical course of the ED may be a consequence of late treatment initiation [41]. However, to our knowledge, this is the first study that also considers it as a barrier. A longstanding ED may delay the start of treatment and perpetuate the illness, as the difficulty of seeking help is accentuated due to the ego-syntonic nature of symptoms.

Only one patient interviewed did not refer to her mother as an important figure in the therapeutic process. This is in accordance with data from the international literature, where fathers are less involved than mothers in treatment [36, 42, 43].

The average DUED estimated in our study was 7.6 years (median 5.8 years), representing, to our knowledge, the longest time published in the literature on ED. The clinical characteristics of the sample could partially explain this finding since this study included patients with different types of EDs. Most publications have been done in patients with AN, which may be identified earlier as the severity is more visible and recognized than others EDs. A recent systematic review found that the DUED for AN was 2.5 years, while for BN it was 4.4 years, and for binge-eating disorder 5.6 years [7]. Another explanation for the long DUED could be the limited availability of specialized ED services in Chile and the unfamiliarity of the Chilean population, including professionals, with EDs. This finding should prompt Chilean authorities to consider new strategies to facilitate healthcare access in EDs. Also, it should prompt national and international studies aiming to estimate DUED in specific populations to establish a comparison framework.

Evidence from Australia and Germany, both of which have robust public healthcare systems, indicates that reducing the DUED remains a significant challenge even in high-resource settings [26, 44]. However, the Australian study found that only a small portion of the DUED was attributable to the referral phase, potentially highlighting an advantage of their healthcare system. As this study suggests, patients with EDs in Chile encounter significant challenges while awaiting appropriate referral and specialized treatment.

After the studies by Kästner and McNicholas [33, 45], this is the third qualitative publication that explores barriers to treatment for patients with EDs from the perspective of patients, family members, and health professionals. To our knowledge, this is the first study done in a developing country. This may contribute to filling an important gap in the literature since all the studies published to date have been conducted in countries with greater resources.

### Limitations

Most of the study limitations are due to the small sample size. Although there was theoretical saturation, three variables could not be explored exhaustively (migrant status, indigenous people, and male gender). Patient gender may be important, as men with EDs are often less recognized, less treated, and more stigmatized [46]. Primary healthcare professionals were not included. Moreover, the health professionals included in the study were mainly ED specialists working in a private clinic, which is uncommon for the Chilean healthcare system. Although few professionals from the public healthcare system were interviewed, the inclusion of specialists mostly from the private system represents the existing gap between the public and private healthcare systems in Chile.

Even though this study includes patients and families from various regions of Chile, it fails to represent the whole country. There is a bias in the patients’ sample, as they were receiving treatment. The literature shows that patients with EDs, particularly young people, often do not seek treatment and that this “untreated” group may manifest other barriers [39, 47]. Second, the sample lacked participants from more extreme regions of the country (such as the Extreme North and Patagonia), which could have further enriched the analysis. Last, there is a significant overrepresentation of patients with private health insurance (68% in the sample versus 16.9% according to official data [48]). Although the high cost of treatment was one of the most relevant barriers in the analysis, the referral processes and the lack of specialists would have had a greater weight if a representative sample of the Chilean public health insurance system had been included.

### Public health implications

Society is relatively poorly educated about EDs, and public education programs are warranted. There is also a lack of education among health professionals in recognizing symptoms and risk factors of ED that delay diagnosis. Interventions at both levels may be a cornerstone for a public health intervention.

Early treatment in EDs is important, considering the early onset age and the neurobiological consequences over time [49]. Some population-level interventions have shown promising results. In England, FREED (First Episode Rapid Early Intervention) is an evidence-based early intervention model for EDs focused on people aged 16 to 25. It has successfully reduced waiting lists, DUED, and clinical outcomes [50, 51]. It has been implemented in 80% of NHS services for adults [52]. FREED has been modified for use in Australia, where Australian Medicare funds treatment up to 40 therapy sessions and 20 dietitian appointments per year, but with a focus on severe ED [53]. In contrast, a German program that sought to reduce the DUED of EDs with a systemic public health intervention, including school activities, failed to show statistical significance, probably due to methodological issues [54].

Cost-effectiveness in developing interventions to prevent and treat EDs has been described when public health systems are involved. An example of this is the English FREED model for intensive treatment of EDs where 4.472 pounds were saved per patient [48]. In Germany, savings of 2 to 4 euros were estimated per euro invested in AN, and 4 euros saved per euro invested in BN [55]. It must be emphasized that in both countries, the public health system is superior to Chile’s.

In Chile, the Explicit Health Guarantees (GES) model mandates a set of benefits that both public and private healthcare providers must offer, ensuring access, quality, financial protection, and timely care for 87 specified diseases or health conditions, with an adherence rate of 84% [56, 57]. Integrating EDs into the GES framework could address major challenges faced by patients, such as timely access to treatment, insurance coverage for ED treatment, while increasing the visibility of EDs and financing the development of prevention and promotion programs across all regions. Reconceptualizing EDs as a major public health issue could facilitate their inclusion in medical education undergraduate programs and the implementation of prevention campaigns in schools. Preventive and therapeutic interventions for EDs have the potential to reduce the economic burden these disorders impose on the healthcare system [58].

### Future research

We suggest conducting more studies on access to treatment for patients with EDs in developing countries. In the long run, low visibility affects the development of public health policies, the physical and mental health of patients, and the perpetuation of stigma.

Moreover, it would be useful to encourage epidemiological studies on EDs in Latin America to favor the visibility of the problem and decision-making. Likewise, it would be worthwhile to inquire more into patients’ experiences within the public system, male patients’ experiences, and stratifying by sexual orientation and ethnic groups. Conducting studies on barriers and facilitators to access in indigenous peoples may contribute to filling an important research gap in eating disorders while providing relevant data on the importance of including those experiences in healthcare services, as recent evidence suggests [38].

Previously, we acknowledged that this study didn’t include individuals with EDs who have not accessed specialist care. However, this would be a key point for future studies regarding access to treatment in developing countries like Chile. Furthermore, individuals diagnosed with an ED in primary care who never entered specialist treatment could be a key target group for understanding barriers and developing public policies.

This study didn’t include primary healthcare professionals. Future research could emphasize in the barriers that primary care face in diagnosing, treating and referring patients with EDs, which could be crucial for public policy development.

## Conclusion

This study provides qualitative information regarding access to treatment for EDs in Chile. It is the first study of its kind done in a developing country. This may favor the understanding of the phenomenon as a global problem.

When dealing with EDs, the entire family unit is distressed. Interventions and proposals should be aimed at modifying family dynamics inside and outside health care to facilitate rapid identification. Likewise, the effect of the barriers inherent to the healthcare system and the inadequate training of professionals should be considered. Finally, it is necessary to reduce DUED to avoid worse clinical outcomes and chronicity of the disorder.

## Supplementary Information

Supplementary Material 1

Supplementary Material 2

## Data Availability

The anonymized interview transcripts will be made available by the authors upon legitimate request.

## Abbreviations

AN: Anorexia nervosa
ARFID: Avoidant/restrictive food intake disorder
BMI: Body-mass-index
BN: Bulimia nervosa
DSM-5: Diagnostic and statistic manual of mental disorders—fifth edition
DUED: Duration of untreated eating disorder
ED: Eating disorder
EDs: Eating disorders
FSE: Family and social environment
HCS: Healthcare system
HP: Health professionals
OSFED: Other specified feeding or eating disorder
P: Patient
SCC: Social and cultural context
UFED: Unspecified feeding or eating disorder
USD: United States dollar
